# Alexander G. Bick receives the 2025 ASCI/Seldin~Smith Award for Pioneering Research

**DOI:** 10.1172/JCI199946

**Published:** 2025-12-15

The American Society for Clinical Investigation (ASCI) honors Alexander G. Bick, MD, PhD (Figure 1), with the 2025 ASCI/Seldin~Smith Award for Pioneering Research. Dr. Bick is recognized for his application of computational genomics and experimental techniques to advancing understanding of blood cancer and cardiovascular disease. He is Director of the Division of Genetic Medicine at Vanderbilt University Medical Center and was elected to the ASCI in 2025. Dr. Bick received his MD and PhD in genetics from Harvard and completed a residency at Massachusetts General Hospital (MGH) and fellowship at the Broad Institute. Rajan Jain, MD, ASCI Councilor and William Wikoff Smith Associate Professor in Cardiovascular Research at the University of Pennsylvania Perelman School of Medicine, interviewed Dr. Bick at the AAP/ASCI/APSA Joint Meeting in Chicago in April 2025.

Rajan Jain: It’s my pleasure to be here with Dr. Alexander Bick, the 2025 Seldin~Smith Award recipient. Dr. Bick, could you tell us about what motivated you to set up your own lab and make research a central focus of the work you’re doing?

Alexander G. Bick: As an undergraduate, I studied bioengineering, and I think that gave me more of a quantitative perspective on life. As I got into medical school, doing clinical rotations, I saw the incredible impact that we were having as physicians on the lives of our patients. Then I thought about it a quantitatively and said, “Let’s say I’m a phenomenally successful physician. I might be able to have an impact on, say, thousands of patients.” Then I thought about it a little more and said, “But if I was a physician-scientist and change not just how I was practicing medicine, but how colleagues around the world could practice medicine, it could have an even greater impact.” So what motivated me to pursue this career, and continues to motivate me every day, is to think about how we can transform medicine in the biggest way possible. That’s what we do in our lab and our clinic. I’m excited to be part of a society like the ASCI where that’s the mission of truly everybody in the community.

RJ: That that resonates with many of us in the Society. As a human geneticist, what do you think are the major questions going forward that need to be tackled or that you’re excited to tackle?

AGB: Human genetics has had a revolution over the past 30 years, as sequencing technologies have become more ubiquitous, cheaper, and are now clinically used every day. When I was a graduate student studying human genetics, I was focused on what made people different from one another. We sequenced individuals from the Framingham Heart Study and found that some went on to get cardiomyopathy, some, coronary artery disease. Why was that? Studying these differences was what drove this wave of human genetics. One of the main principles was that your genome doesn’t change: the genome that you’re born with is the genome you have for the rest of your life. What I came to discover when I was doing my postdoctoral work with Sek Katherisen, in collaboration with many other ASCI members like Peter Libby and Benjamin Ebert, was that your genome does change over the course of your lifetime. That not only [are there] differences between people in their genomes, but inside every single cell in our body, our genomes are slightly different. This new dimension, this new complexity is what’s been a key focus of my group since we started five years ago and continues to be. We discovered that people have mutations in their blood that they’re not born with. These not only cause diseases like cancer, but also contribute to heart disease and other diseases of inflammation in strange and surprising ways. That’s where we’ve been.

Where are we going? I think there are two different directions. One is that we’ve studied blood, because we have a million blood genomes to study. If all of the biobanks in the world were collecting cheek swabs, we would perhaps know a lot more about the oral epithelium. But we’ve developed tools along the way to study blood that we are now able to take into many different systems in the body. From our early observations, just like the blood, there are these different mutations in every dividing cell type. Understanding these mutations and what they do is really exciting. But it’s not just enough to understand. Our goal as physician-scientists is to make an impact. One thing that’s been incredible for me as an early-career physician-scientist is seeing how quickly that can happen. This process of these mutant blood cells was first described in 2014, about 10 years ago. Just this year, we finished the first phase II clinical trials enrolling patients on the basis of having these mutant blood cells. To be able to go from observation all the way to human impact in a decade is absolutely unbelievable and something that we’re continuing to push forward in both the lab and the clinic.

RJ: Echoing some of the things you were saying, I think bringing all the new single-cell technologies and imaging technologies to bear on somatic mosaicism will lead to a fundamental revolution in our understanding of human genetics and, like you mentioned, the ability to intervene at early time points. Taking a step back, could you tell us about some of the obstacles that you had to overcome on this journey that you’ve been on, first in Boston and now at Vanderbilt?

AGB: One of the challenges has been that I’ve always wanted to do both things all the time. Even when I was a first-year medical student taking anatomy at nights, on the weekends, I was going back to the lab to try and keep my cell culture alive. I actually almost failed the first-year anatomy class. My physician-scientist career almost ended right then and there. This has been a recurring theme throughout my career. Then I finished the PhD. It went really well, and I wanted to go into residency. Again, in residency I wanted to keep my research going because the field was moving so quickly that I didn’t want to leave it for five years, eight years, and come back. I’ve always been extraordinarily lucky to have not just mentors, but sponsors, to have people who have tried to imagine with me how to make that goal possible. When I was interviewing for medicine residency at MGH, our chair of medicine, Katrina Armstrong, program director, Jay Vyas, and Physician-Scientist Pathway director, Jay Rajagopal, said, “We’ve never done anything like this before, but the first intern year, you should learn how to be a doctor. So the first nine months, let’s do clinical work. But after that, you can take three months and do clinical work, three months and do research, and go back and forth.” Doing that dramatically accelerated what I was able to do during residency. I did four years of medicine residency, but I also did four years of postdoc at the same time. At the end of that, I had enough of a research program developed that I was able to just start my own lab. Imagining how we make these audacious requests possible is always a challenge. Again, I’ve been so fortunate to have physician-scientists in my life to have made that possible.

RJ: It seems like you’ve had great sponsors and mentors who allowed you to think flexibly about what was best for your path. I know that conversation is happening across the nation as we’ve been trying to think about how to decrease the time necessary for training. You were recently named director of Genomic Medicine at Vanderbilt. Could you talk about how you balance the demands for running a successful research lab and spending time in clinic — and now administrative responsibilities.

AGB: As a trainee, we had lots of great luminaries talk about how they balance their research and clinical work. I imagined that would be really challenging. But then we had three children, and my wife is a neurosurgeon, so that makes me the flexible spouse in the family. On some days I wake up in the morning, and the challenge of balancing being a physician and a scientist pales in comparison to the challenge of being a dad and getting the kids to all their after-school activities. But on a more serious note, the model that I’ve tried to follow, which I’ve learned from many mentors, is to try and make the two as synergistic as possible. My clinic in genomic medicine is very well circumscribed. People generally don’t have after-hour emergencies. Their genome might change, but it doesn’t change that much, that quickly. In adult medicine and adult genetics, there are a lot fewer emergencies. [Maintaining a balance also involves] making sure, in collaboration with leadership, that clinical work is well supported, that I have the genetic counselors to make sure the results are followed up on, even if I’m a little slow to do that. Having the right support certainly helps. But then [it’s] not just the support, but the work that I’m doing in the clinic, seeing patients with clonal hematopoiesis with these mutant blood cells, seeing patients with some of the same genetic alterations that we’re studying in the lab, being able to take samples from those patients back to the lab. So when I’m in the clinic, everything that I’m doing in the lab is making me a better doctor for those patients. I’m certainly nowhere near as good a doctor as I was at the end of residency in terms of being a general internist, but I think that I do a good enough job for the patients I care for. And administratively now, when I had the opportunity to take on leading genetics in the Department of Medicine at Vanderbilt, it was the same motivation: “How do we take what we’re learning in our research lab and make sure that all of our adult medicine patients are really benefiting from personalized medicine?” The opportunity to have that seat at the table alongside all the other division directors in the department — and institutionally now with leadership of our biobank — and make sure that what we’re doing is benefiting all of our patients as quickly as possible has been exciting.

RJ: You’ve been fortunate to have many mentors, supporters, and sponsors along the way and still do. How can we best encourage and support young physician-scientists? I noticed you have multiple MD-PhD students, multiple fellows in your lab at differing stages.

AGB: Just like we’re trying to make the care of all our patients personal, we also have to think as the senior leaders of American biomedical research about “How can we take each individual and make their pathway work the best for them?” I mentioned earlier, how in residency, MGH created a pathway that didn’t exist before for me. Subsequently, they’ve had others who have been interested in it, though this is not the best or the only way for everyone. But just to have the imagination that things can be different and that we can take what one of our trainees is telling us they want to do and make that possible is a unique opportunity for all of us. And then study that variation. We are all scientists after all. If we create an individualized pathway, can we say, “Is that reproducible? Were the outcomes any different?” Obviously, that is a hard challenge, but I think one that we’re all up to.

RJ: Thank you so much for joining us and sharing these great thoughts, and congratulations again on being awarded the 2025 Seldin~Smith Award.

AGB: Thank you so much.

*The interview has been edited for length and clarity*.

## Figures and Tables

**Figure 1:**
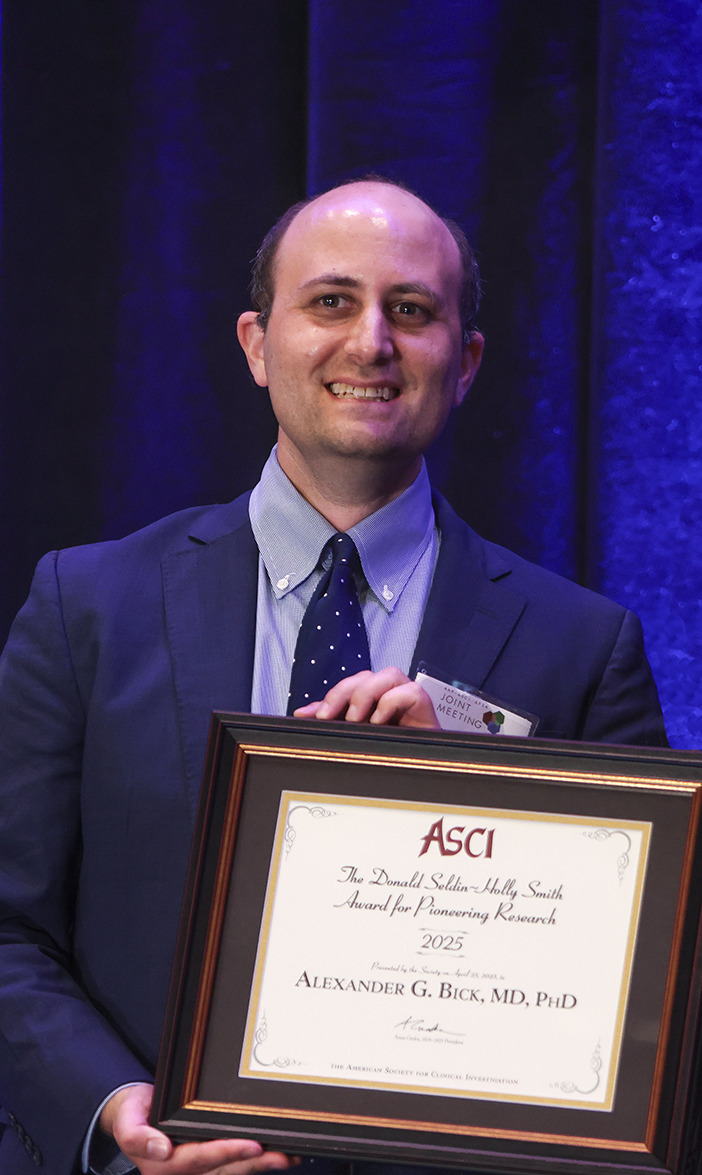
Alexander G. Bick is the recipient of the 2025 Seldin~Smith Award for Pioneering Research.

## Version 1 12/15/2025

Electronic publication

